# Common Secondary Genomic Variants Associated With Advanced Epithelioid Hemangioendothelioma

**DOI:** 10.1001/jamanetworkopen.2019.12416

**Published:** 2019-10-02

**Authors:** Nathan D. Seligson, Achal Awasthi, Sherri Z. Millis, Brian K. Turpin, Christian F. Meyer, Anne Grand'Maison, David A. Liebner, John L. Hays, James L. Chen

**Affiliations:** 1 The Ohio State University Wexner Medical Center and Comprehensive Cancer Center, The Ohio State University, Columbus; 2 Department of Biomedical Informatics, The Ohio State University, Columbus; 3 Foundation Medicine Inc, Cambridge, Massachusetts; 4 Division of Pediatric Hematology/Oncology, Cincinnati Children's Hospital Medical Center, Cincinnati, Ohio; 5 Division of Medical Oncology, Johns Hopkins Medical Center, Baltimore, Maryland; 6 Department of Medical Oncology, Roswell Park Cancer Center, Buffalo, New York; 7 Division of Medical Oncology, Department of Internal Medicine, The Ohio State University, Columbus; 8 Division of Gynecologic Oncology, Department of Obstetrics and Gynecology, The Ohio State University, Columbus

## Abstract

**Importance:**

Epithelioid hemangioendothelioma (EHE) is a rare, malignant vascular sarcoma characterized in most cases by a *WWTR1*-*CAMTA1* fusion. The clinical course of EHE exhibits a dual nature. The condition is often indolent but can rapidly grow and metastasize unpredictably. No biomarkers to date are available to predict this phenotype. The hypothesis of this study was that better defining the genomic landscape of EHE using next-generation sequencing could offer additional therapies and insight into clinical outcomes.

**Objective:**

To characterize secondary EHE genomic alterations and their association with clinical outcomes.

**Design, Setting, and Participants:**

Multicenter, cross-sectional, retrospective study of next-generation sequencing results collected from participants diagnosed with EHE. Data were abstracted between May 1, 2013, and May 31, 2019. This analysis was conducted from January through June 2019. Summary genomic data were provided by commercial genomic testing companies.

**Main Outcomes and Measures:**

Presence or absence of secondary pathogenic genomic variants and their association with disease stage and clinical features.

**Results:**

A total of 49 participants with EHE were assessed for the presence or absence of secondary genomic variants. Of these, 32 (65.3%) were female; the mean (SD) age at diagnosis was 49.9 (18.3) years (range, 11-81 years). In all, 46 participants (93.9%) had confirmed *WWTR1-CAMTA1* fusion; 26 participants (57.1%) exhibited a pathogenic genomic variant secondary to the *WWTR1-CAMTA1* fusion; and 9 participants (18.4%) exhibited potentially targetable genomic variants. Commonly altered genes included *CDKN2A/B*, *RB1*, *APC*, and *FANCA*. Participants older than 45 years at diagnosis had an increased prevalence of secondary genomic variants that was not statistically significant (65.6% vs 38.5%; difference, 27.1%; 95% CI, −3.5% to 58.0%; *P* = .16) and were more likely to have a clinically targetable variant (28.1% vs 0%; difference, 28.1%; 95% CI, 11.2%-40.2%; *P* = .03). In 14 participants with clinical data available, those with stage III/IV EHE were more likely to exhibit a secondary pathogenic genomic variant (80% vs 0%; difference, 80%; 95% CI, 55.2%-100%; *P* = .006). Participants with stage III/IV EHE were diagnosed at an older age (mean [SD] age, 54.6 [14.1] years vs 31.7 [16.0] years; *P* = .05) and had elevated *WWTR1-CAMTA1* fusion expression that was not statistically significant (mean [SD] expression, 677 [706] copies vs 231 [213] copies; *P* = .20).

**Conclusions and Relevance:**

Although EHE exhibits few secondary genomic variants, presence of key secondary variants may be prognostic for aggressive EHE. Further research is needed to confirm this finding and determine whether more intensive upfront treatment is necessary for these patients.

## Key Points

**Question:**

Can next-generation sequencing reveal rationale for the dichotomous biological activity of epithelioid hemangioendothelioma (EHE) while illuminating potentially actionable alterations?

**Findings:**

In a cross-sectional study of next-generation sequencing results collected from 49 participants diagnosed with EHE, more than half of patients with EHE profiled exhibited pathogenic genomic variants in addition to the *WWTR1-CAMTA1* fusion, with 18.4% of participants exhibiting a potentially targetable variant. Participants with stage III/IV EHE were more likely to exhibit a secondary pathogenic variant.

**Meaning:**

Next-generation sequencing may identify secondary genomic variants that are associated with EHE aggressiveness; additionally, these variants may represent potential therapeutic targets.

## Teaser

This cross-sectional study characterizes secondary genomic alterations in patients with epithelioid hemangioendothelioma and their association with clinical outcomes.

## Introduction

Epithelioid hemangioendothelioma (EHE) is a rare vascular sarcoma with a prevalence of approximately 1 per 1 000 000 persons.^1^ A hallmark molecular characteristic of EHE is the fusion of the *WWTR1* and *CAMTA1* genes, present in 90% of EHE cases and pathognomonic for disease.^2,3,4^ The clinical course of EHE may be either indolent (often locally limited) or aggressive (characterized by local invasiveness or metastasis); however, indolent disease can unpredictably become aggressive. Key molecular biomarkers indicative of EHE course have yet to be established^5^; however, mitotic count and tumor size have been associated with prognosis.^6^ While limited disease can be amenable to observation or local therapy, metastatic EHE is typically resistant to chemotherapy and carries a poor prognosis. Treatment for advanced-stage EHE is not well established.^7^ Pathway-specific targeted therapies hold some promise, but improved systemic therapies are still needed.^8^

Few reports describe the genomic landscape of EHE outside of the driver fusions with their clinical correlates and have described a mostly quiet genome.^9^ In this article, we present the largest assessment, to our knowledge, of the clinicogenomic landscape of *WWTR1-CAMTA1* (*WC*) fusion–associated EHE.

## Methods

Data were abstracted between May 1, 2013, and May 31, 2019. This analysis was conducted from January through June 2019. Summary genomic data was provided by commercial genomic testing companies. This study is reported in accordance with the Strengthening the Reporting of Genetic Association Studies (STREGA) reporting guideline.^10^

### Retrospective Analysis

Approval for the retrospective collection of genomic data from Foundation Medicine, including a waiver of informed consent and HIPAA waiver of authorization, was obtained from the Western Institutional Review Board. Participants diagnosed with EHE were identified from retrospective sarcoma studies at The Ohio State University James Comprehensive Cancer Center, Roswell Park Cancer Institute, Johns Hopkins Medical Center, and Cincinnati Children's Hospital Medical Center. Waiver of informed consent for the original studies was approved by local institutional review boards. Participant characteristics, tumor stage at time of biopsy, and genomic data were extracted for this study. All participants identified were included. Sample size was based on data available, and no sample size calculations were performed.

### Genomic Analysis

Genomic profiling data were collected from 46 patients with EHE who underwent genomic sequencing by Foundation Medicine (FMI)^11^ and 3 who underwent genomic sequencing by OmniSeq.^12^ Participants’ *WC* fusion status was only confirmed for those profiled by FMI. The FMI FoundationOne Heme panel includes coverage of 426 fully sequenced genes, rearrangement of 32 genes, and fusions of 282 genes. The OmniSeq panel includes coverage of 26 fully sequenced genes, hot spots in 73 genes, copy number variants in 52 genes, and fusions of 23 genes. Full genomic coverage of both targeted next-generation platforms is outlined in eTable 1 in the Supplement. Pathogenicity of genomic variants for participants sequenced by OmniSeq was determined via the COSMIC database.^13^

Pathogenic variants and variants of unknown significance (VUS) were included in our analysis. Genomic variants identified apart from the *WC* fusion were considered secondary variants. Gene enrichment was performed using Superpaths^14^ (eTable 2 in the Supplement). Targetable variants were defined using OncoKB classification as previously described.^15^

### Statistical Analysis

All data were analyzed in R statistical software version 3.4.3 (R Project for Statistical Computing) or Prism analysis and graphing software version 8.0.0 (GraphPad). For continuous variables, *t* tests were used. For categorical variables, χ^2^ tests were used to generate *P* values and a test of proportions was used to generate 95% confidence intervals of proportion difference. Continuous data are presented as mean (SD) unless otherwise stated and 2-tailed *P* values ≤.05 were considered statistically significant.

## Results

### Patient Characteristics

Of 49 participants with EHE analyzed (32 [65.3%] female; mean [SD] age at diagnosis, 49.9 [18.3] years [range, 11-81 years]), 46 (93.9%) had *WC* fusion confirmation. These participants were primarily female (29 patients [63.0%]), and the mean (SD) age at diagnosis was 50.2 (18.5) years (range, 11-81 years). Full demographic characteristics are available in the Table. Participants had a low tumor mutation burden (mean [SD] mutations per megabase, 1.1 [1.5]). Quantification of *WC* expression from available participants demonstrated a right-skewed, log-normal distribution (eFigure 1A and B in the Supplement).

**Table.** Demographic Characteristics

Characteristic	No. (%)		
	*WWTR1-CAMTA1* Fusion (n = 46)	No *WWTR1-CAMTA1* Fusion (n = 3)	Total (N = 49)
Age at diagnosis, mean (SD) [range], y	50.2 (18.5) [11-81]	45.3 (18.7) [25-62]	49.9 (18.3) [11-81]
Sex			
Male	17 (37.0)	0	17 (34.7)
Female	29 (63.0)	3 (100)	32 (65.3)
Microsatellite status			
Microsatellite stable	32 (70.0)	0	32 (65.3)
Not tested	14 (30.0)	3 (100)	17 (34.7)
Tumor mutation burden, mean (SD), mutations per megabase	1.1 (1.5)	ND	1.1 (1.5)
Pathogenic genomic variants			
Data not available	0	1 (33.3)	1 (2.0)
*WWTR1-CAMTA1* only	20 (43.5)	0	20 (40.8)
Additional variants, No.			
1	14 (30.4)	0	14 (28.6)
2	9 (19.5)	1 (33.3)	10 (20.4)
≥3	3 (6.6)	1 (33.3)	4 (8.2)

Abbreviation: ND, no data.

### EHE and Secondary Alteration in Established Oncogenic Pathways

**Figure 1.**
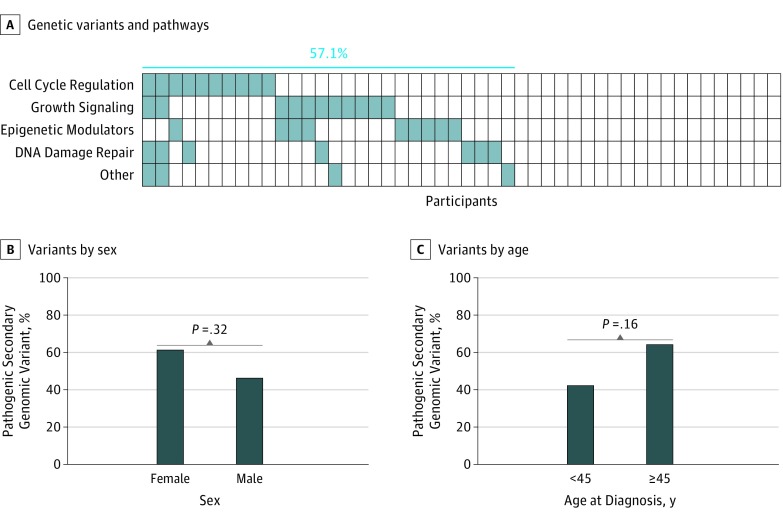
Genomic Landscape of Epithelioid Hemangioendothelioma A, Heatmap of the presence (shaded) or absence (white) of known genomic variants based on their shared pathways. While a majority of epithelioid hemangioendothelioma tumors demonstrated a secondary genomic variant (57.1%), few tumors exhibited genomic variants in multiple pathway categories. B, Pathogenic secondary genomic variants were more common in female participants, but the difference was not statistically significant. C, Pathogenic secondary genomic variants were also more common in participants aged 45 years or older at diagnosis, but the difference was not statistically significant.

In all, 21 participants with EHE (42.9%) exhibited a *WC* fusion as a sole pathogenic genomic variant. A single additional pathogenic variant was identified in 14 participants (28.6%), while 2 or more pathogenic variants were identified in an additional 14 participants. The most commonly identified secondary variants were seen in *CDKN2A* (6 pathogenic, 1 VUS), *CDKN2B* (4 pathogenic, 0 VUS), *RB1* (2 pathogenic, 1 VUS), *ATRX* (2 pathogenic, 1 VUS), *APC* (2 pathogenic, 1 VUS), and *FANCA* (2 pathogenic, 0 VUS) (eTable 3 in the Supplement). Pathways identified as altered in EHE included cell cycle regulation, growth signaling, epigenetic modulators, and DNA damage repair (Figure 1A). Twenty-six participants (57.1%) exhibited a pathogenic genomic variant secondary to the *WC* fusion, and 9 participants (18.4%) exhibited genomic variants in multiple pathways. Commonly altered genes included *CDKN2A/B*, *RB1*, *APC*, and *FANCA*. Sex did not segregate secondary genomic variant frequency (47% male vs 62% female; difference, 15%; 95% CI, 13.6%-44.5%; *P* = .32) (Figure 1B).

### Older Age and Increased Genomic Complexity

Age at diagnosis demonstrated a bimodal distribution with a division at 45 years (log-likelihood 1-component model, −207.2 vs 2-component model, −197.6; difference, 9.6; 95% CI, 0.0-23.8; *P* = .02) (eFigure 2 and eMethods in the Supplement). Participants aged 45 years or older at diagnosis had a higher prevalence of pathogenic secondary genomic variants that was not statistically significant (65.6% vs 38.5%; difference, 27.1%; 95% CI, −3.5% to 58.0%; *P* = .16) (Figure 1C). Notably, variants in the most commonly altered gene in this data set, *CDKN2A,* were exclusively seen in participants aged 45 years or older at diagnosis (eTable 4 in the Supplement).

**Figure 2.**
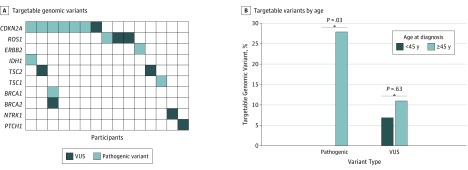
Targetable Genomic Variants in Epithelioid Hemangioendothelioma A, Heatmap of the presence of targetable pathogenic genomic variants (dark blue), targetable variants of unknown significance (VUS) (light blue), or the absence of targetable genomic variants (white) as defined by OncoKB. B, Participants aged 45 years or older at diagnosis were more likely to exhibit a targetable pathogenic genomic variant, but equally likely to demonstrate a targetable VUS compared with participants younger than 45 years at diagnosis.

A total of 19 targetable variants were identified by OncoKB (Figure 2A; eTable 5 in the Supplement). Pathogenic genomic variants identified to be targetable were seen in 9 participants (18.4%), with 5 participants (10.2%) harboring variants associated with US Food and Drug Administration–approved therapies. Participants aged 45 years or older at diagnosis were more likely to have a targetable pathogenic genomic variant (28.1% vs 0%; difference, 28.1%; 95% CI, 11.2%-40.2%; *P* = .03) (Figure 2B).

### Presence of Secondary Alterations and Advanced-Stage Disease

**Figure 3.**
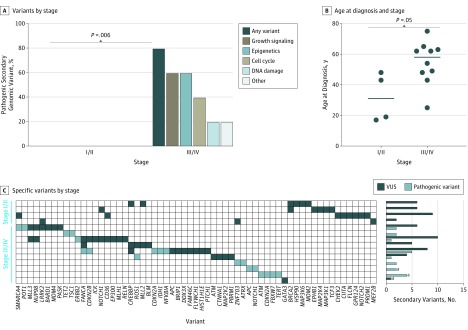
Prevalence of Pathogenic Secondary Genomic Variants A, Stage III/IV tumors were more likely to harbor a pathogenic secondary genomic variant. B, Stage III/IV tumors were associated with older age at diagnosis. Horizontal lines indicate group median. C, Heatmap of secondary genomic variants with total secondary variants noted on the rightmost y-axis. VUS indicates variant of unknown significance. ^a^Three participants clinically diagnosed with stage III/IV epithelioid hemangioendothelioma with genomic profiling data available but lacking confirmation of a *WWTR1-CAMTA1* fusion were included in a secondary clinical assessment. These participants exhibited similar characteristics to other participants previously described.

To assess the clinicogenomic landscape of EHE, 14 participants with clinical data available were identified (4 with stage I/II, 10 with stage III/IV). Participants with stage III/IV EHE were significantly more likely to exhibit a pathogenic secondary genomic variant (80% vs 0%; difference, 80%; 95% CI, 55.2%-100%; *P* = .006) (Figure 3A and C). Additionally, those with stage III/IV EHE were older at diagnosis (mean [SD] age, 54.6 [14.1] years vs 31.7 [16.0] years; *P* = .05) (Figure 3B) and had greater *WC* fusion expression that was not statistically significant (mean [SD], 677 [706] vs 231 [213] copies; *P* = .20) (eFigure 1C in the Supplement).

## Discussion

Epithelioid hemangioendothelioma is characterized molecularly by its primary gene fusions, but owing to the rarity of this disease, little is known regarding the clinical significance of secondary genomic alterations. Here, we present the largest assessment, to our knowledge, of the genomic landscape of *WC* fusion–positive EHE. Of the 49 participants included in this study, 46 were positively identified to have the *WC* fusion with no additional fusions detected. An additional 3 participants with EHE were included after histopathological review. The less common EHE fusion, *YAP1-TFE3*,^16^ was not explicitly tested for by the OmniSeq panel. No participants tested with the FMI panel were identified to have concurrent *WC* and *YAP1-TFE3* fusions. We have included this OmniSeq data as well, given the high likelihood of a *WC* fusion.

Although half of all EHE tumors included in this study exhibited a pathogenic secondary genomic variant, it was rare for a tumor to have 2 or more secondary variants present. The identified variants are linked to well-studied oncogenic pathways. The most prevalent gene alteration identified in this study was deletion of the *CDKN2A/B* locus, corresponding to well-studied tumor suppressor genes responsible for regulation of the cell cycle and p53-mediated apoptosis. The data available here are unable to test the importance of *CDKN2A/B* loss in the natural history or development of EHE. Further study is necessary to identify the role of *CDKN2A/B* loss in EHE. In other sarcomas, including gastrointestinal stromal tumors, loss of *CDKN2A* expression is associated with poor prognosis and a greater potential for metastatic disease.^17,18,19^ The biological meaning of *CDKN2A/B* loss in EHE requires further elucidation.

Approximately 20% of EHEs studied exhibited a clinically actionable secondary genomic alteration. Further assessment identified an enriched prevalence in participants aged 45 years or older at diagnosis. In our clinically enriched subset, stage III/IV EHE was strongly associated with the presence of pathogenic secondary genomic variants and older age. Importantly, this was true when either including or excluding participants lacking confirmation of the *WC* fusion. Taken together, these data suggest that the fusion event may represent the first step in the development of EHE with a secondary genomic change required for tumor aggressiveness. This has several potential practice implications: for one, participants with newly diagnosed EHE could be considered for genomic profiling to evaluate the presence or absence of secondary alterations; in addition, participants with EHE with secondary alterations may potentially be considered for more aggressive treatment. Prospective clinical trials will need to confirm this guidance.

### Limitations

To our knowledge, this is the largest genomic assessment of EHE to date; however, limitations inherent to studies of extremely rare diseases apply here. As tumor sequencing is not a standard recommendation in the treatment of EHE, the data available may suffer from selection bias toward more aggressive EHE. Additionally, the 2 next-generation sequencing platforms used to test for genomic variants are targeted to a specific set of genes and vary significantly in their coverage. Whole-genome sequencing approaches may provide a more comprehensive assessment; however, the targeted panels used here provide strong, validated assessment of genes known to have biological and clinical associations with cancer. It is important to note that participants who underwent sequencing by FMI and OmniSeq exhibited similar trends in association between genomic variants and tumor stage. Secondary genomic variants are certainly important in EHE; however, the prevalence of these alterations may be lower in a prospectively curated data set. Additionally, this data set is limited temporally and is unable to differentiate between passenger and active genomic variants. Further longitudinal research is necessary to define the genomic progression of EHE.

## Conclusions

In this study, more than half of participants with EHE and *WWTR1-CAMTA1* fusion exhibited a secondary genomic variant, with up to 20% that are potentially clinically actionable. Participants with advanced-stage EHE were significantly more likely to have secondary genomic variants. Prospective, multigroup clinical trials are necessary to confirm these findings and their clinical utility.

## Notes

Supplement. **eTable 1.** Genomic Coverage of Next-Generation Sequencing Platform **eTable 2.** Description of Pathways **eFigure 1.** Distribution of Genomic *WWTR1-CAMTA1* Fusion Expression **eTable 3.** Gene Variants in EHE **eFigure 2.** Age at Diagnosis **eMethods.** Models **eTable 4.** Clinicogenomic Features of *CDKN2A/B* Variant Subjects **eTable 5.** Targetable Genomic Variants
