# Supplementary material for: Common Secondary Genomic Variants Associated With Advanced Epithelioid Hemangioendothelioma
Source: JAMA Netw Open. 2019 Oct 2;2(10):e1912416. doi: 10.1001/jamanetworkopen.2019.12416 (PMC6777396; doi:10.1001/jamanetworkopen.2019.12416)
Supplement: Supplement. — eTable 1. Genomic Coverage of Next-Generation Sequencing Platform eTable 2. Description of Pathways eFigure 1. Distribution of Genomic WWTR1-CAMTA1 Fusion Expression eTable 3. Gene Variants in EHE eFigure 2. Age at Diagnosis eMethods. Models eTable 4. Clinicogenomic Features of CDKN2A/B Variant Subjects eTable 5. Targetable Genomic Variants [file jamanetwopen-2-e1912416-s001.pdf]

Supplementary Online Content

Seligson ND, Awasthi A, Millis SZ, et al. Common secondary genomic variants associated with advanced epithelioid hemangioendothelioma. *JAMA Netw Open*. 2019;2(10):e1912416. doi:10.1001/jamanetworkopen.2019.12416

eTable 1. Genomic Coverage of Next-Generation Sequencing Platform

eTable 2. Description of Pathways

eFigure 1. Distribution of Genomic *WWTR1-CAMTA1* Fusion Expression

eTable 3. Gene Variants in EHE

eFigure 2. Age at Diagnosis

eMethods. Models

eTable 4. Clinicogenomic Features of *CDKN2A/B* Variant Subjects

eTable 5. Targetable Genomic Variants

This supplementary material has been provided by the authors to give readers additional information about their work.

© 2019 Seligson ND et al. *JAMA Network Open.*

eTable 1: Genomic Coverage of Next-Generation Sequencing Platform

etable 1: Genomic Coverage of Next-Generation Sequencing Platforms	
OMNISEQ ADVANCE	FOUNDATIONONE HEME
Single Nucleotide Variants (SNVs), Insertions, Deletions, and Indels	Full Coding Sequence
Hotspot	*ABL1, ACTB, AKT1, AKT2, AKT3, ALK, AMER1, (FAM123B, or, WTX), APC, APH1A, AR, ARAF, ARFRP1, ARHGAP26, (GRAF), ARID1A, ARID2, ASMTL, ASXL1, ATM, ATR, ATRX, AURKA, AURKB, AXIN1, AXL, B2M, BAP1, BARD1, BCL10, BCL11B, BCL2, BCL2L2, BCL6, BCL7A, BCOR, BCORL1, BIRC3, BLM, BRAF, BRCA1, BRCA2, BRD4, BRIP1, (BACH1), BRSK1, BTG2, BTK, BTLA, C11, orf30, (EMSY), CAD, CALR, CARD11, CBFB, CBL, CCND1, CCND2, CCND3, CCNE1, CCT6B, CD22, CD274, (PDL1), CD36, CD58, CD70, CD79A, CD79B, CDC73, CDH1, CDK12, CDK4, CDK6, CDK8, CDKN1B, CDKN2A, CDKN2B, CDKN2C, CEBPA, CHD2, CHEK1, CHEK2, CIC, CIITA, CKS1B, CPS1, CREBBP, CRKL, CRLF2, CSF1R,*
ABL1, AKT1, ALK, AR, ARAF, BRAF, BTK, CBL, CDK4, CHEK2, CSF1R, CTNNB1, DDR2, DNMT3A, EGFR, ERBB2, ERBB3, ERBB4, ESR1, EZH2, FGFR1, FGFR2, FGFR3, FLT3, FOXL2, GATA2, GNA11, GNAQ, GNAS, HNF1A, HRAS, IDH1, IDH2, IFITM1, IFITM3, JAK1, JAK2, JAK3, KDR, KIT, KNSTRN, KRAS, MAGOH, MAP2K1, MAP2K2, MAPK1, MAX, MED12, MET, MLH1, MPL, MTOR, MYD88, NFE2L2,	

© 2019 Seligson ND et al. *JAMA Network Open.*

NPM1, NRAS, PAX5, PDGFRA, PIK3CA, PPP2R1A, PTPN11, RAC1, RAF1, RET, RHEB, RHOA, SF3B1, SMO, SPOP, SRC, STAT3, U2AF1, XPO1	*CSF3R, CTCF, CTNNA1, CTNNB1, CUX1, CXCR4, DAXX, DDR2, DDX3X, DNM2, DNMT3A, DOT1L, DTX1 ,DUSP2, DUSP9, EBF1, ECT2L, EED, EGFR, ELP2, EP300, EPHA3, EPHA5, EPHA7, EPHB1, ERBB2, ERBB3, ERBB4, ERG, ESR1, ETS1, ETV6, EXOSC6, EZH2, FAF1, FAM46C, FANCA, FANCC, FANCD2, FANCE, FANCF, FANCG, FANCL, FAS, (TFRSF6), FBXO11, FBXO31, FBXW7, FGF10, FGF14, FGF19, FGF23, FGF3, FGF4, FGF6, FGFR1, FGFR2, FGFR3, FGFR4, FHIT, FLCN, FLT1, FLT3, FLT4, FLYWCH1, FOXL2, FOXO1, FOXO3, FOXP1, FRS2, GADD45B, GATA1, GATA2, GATA3, GID4, (C17orf39), GNA11, GNA12, GNA13, GNAQ, GNAS, GPR124, GRIN2A, GSK3B, GTSE1, HDAC1, HDAC4, HDAC7, HGF, HIST1H1C, HIST1H1D, HIST1H1E, HIST1H2AC, HIST1H2AG, HIST1H2AL, HIST1H2AM, HIST1H2BC, HIST1H2BJ, HIST1H2BK, HIST1H2BO, HIST1H3B, HNF1A, HRAS, HSP90AA1, ICK, ID3, IDH1, IDH2, IGF1R, KBKE, IKZF1, IKZF2, IKZF3, IL7R, INHBA, INPP4B, INPP5D, (SHIP), IRF1, IRF4, IRF8, IRS2, JAK1, JAK2, JAK3, JARID2, JUN, KAT6A,*
Full Coding Sequence	
APC, ATM, BAP1, BRCA1, BRCA2, CDH1, CDKN2A, FBXW7, GATA3, MSH2, NF1, NF2, NOTCH1, PIK3R1, PTCH1, PTEN, RB1, SMAD4, SMARCB1, STK11, TET2, TP53, TSC1, TSC2, VHL, WT1	

© 2019 Seligson ND et al. *JAMA Network Open.*

	(MYST3), KDM2B, KDM4C, KDM5A, KDM5C, KDM6A, KDR, KEAP1, KIT, KLHL6, KMT2A, (MLL), KMT2C, (MLL3), KMT2D, (MLL2), KRAS, LEF1, LRP1B, LRRK2, MAF, MAFB, MAGED1, MALT1, MAP2K1, MAP2K2, MAP2K4, MAP3K1, MAP3K14, MAP3K6, MAP3K7, MAPK1, MCL1, MDM2, MDM4, MED12, MEF2B, MEF2C, MEN1, MET, MIB1, MITF, MKI67, MLH1, MPL, MRE11A, MSH2, MSH3, MSH6, MTOR, MUTYH, MYC, MYCL, (MYCL1), MYCN, MYD88, MYO18A, NCOR2, NCSTN, NF1, NF2, NFE2L2, NFKBIA, NKX2-1, NOD1, NOTCH1, NOTCH2, NPM1, NRAS, NT5C2, NTRK1, NTRK2, NTRK3, NUP93, NUP98, P2RY8, PAG1, PAK3, PALB2, PASK, PAX5, PBRM1, PC, PCBP1, PCLO, PDCD1, PDCD11, PDCD1LG2, (PDL2), PDGFRA, PDGFRB, PDK1, PHF6, PIK3CA, PIK3CG, PIK3R1, PIK3R2, PIM1, PLCG2, POT1, PPP2R1A, PRDM1, PRKAR1A, PRKDC, PRSS8, PTCH1, PTEN, PTPN11, PTPN2, PTPN6, (SHP-1), PTPRO, RAD21, RAD50, RAD51, RAF1, RARA, RASGEF1A, RB1, RELN, RET, RHOA, RICTOR, RNF43, ROS1, RPTOR, RUNX1, S1PR2, SDHA,SDHB,

© 2019 Seligson ND et al. *JAMA Network Open.*

	SDHC, SDHD, SERP2, SETBP1, SETD2, SF3B1, SGK1, SMAD2, SMAD4, SMARCA1, SMARCA4, SMARCB1, SMC1A, SMC3, SMO, SOCS1, SOCS2, SOCS3, SOX10, SOX2, SPEN, SPOP, SRC, SRSF2, STAG2, STAT3, STAT4, STAT5A, STAT5B, STAT6, STK11, SUFU, SUZ12, TAF1, TBL1XR1, TCF3, (E2A), TCL1A, (TCL1), TET2, TGFBR2, TLL2, TMEM30A, TMSB4XP8, (TMSL3), TNFAIP3, TNFRSF11A, TNFRSF14, TNFRSF17, TOP1, TP53, TP63, TRAF2, TRAF3, TRAF5, TSC1, TSC2, TSHR, TUSC3, TYK2, U2AF1, U2AF2, VHL, WDR90, WHSC1, (MMSET, or, NSD2), WISP3, WT1, XBP1, XPO1, YY1AP1, ZMYM3, ZNF217, ZNF24, (ZSCAN3), ZNF703, ZRSR
Copy Number Gain	DNA Rearrangements
ACVRL1, AKT1, APEX1, AR, ATP11B, BCL2L1, BCL9, BIRC2, BIRC3, CCND1, CCNE1, CD274, CD44, CDK4, CDK6, CSNK2A1, DCUN1D1, EGFR, ERBB2,	*ALK, BCL2, BCL6, BCR, BRAF, CCND1, CRLF2, EGFR, EPOR, ETV1, ETV4, ETV5, ETV6, EWSR1, FGFR2, IGH, IGK, IGL, JAK1, JAK2, KMT2A, (MLL), MYC, NTRK1, PDGFRA, PDGFRB, RAF1, RARA, RET, ROS1, TMPRSS2, TRG*

© 2019 Seligson ND et al. *JAMA Network Open.*

FGFR1, FGFR2, FGFR3, FGFR4, FLT3, GAS6, IGF1R	
Copy Number Loss	Gene Fusions
APC, ATM, BAP1, BRCA1, BRCA2, CDH1, CDKN2A, FBXW7, GATA3, MSH2, NF1, NF2, NOTCH1, PIK3R1, PTCH1, PTEN, RB1, SMAD4, SMARCB1, STK11, TET2, TP53, TSC1, TSC2, VHL, WT1	*ABI1, ABL1, ABL2, ACSL6, AFF1, AFF4, ALK, ARHGAP26, (GRAF), ARHGEF12, ARID1A, ARNT, ASXL1, ATF1, ATG5, ATIC, BCL10, BCL11A, BCL11B, BCL2, BCL3, BCL6, BCL7A, BCL9, BCOR, BCR, BIRC3, BRAF, BTG1, CAMTA1, CARS, CBFA2T3, CBFB, CBL, CCND1, CCND2, CCND3, CD274, (PDL1), CDK6, CDX2, CHIC2, CHN1, CIC, CIITA, CLP1, CLTC, CLTCL1, CNTRL, (CEP110), COL1A1, CREB3L1, CREB3L2, CREBBP, CRLF2, CSF1, CTNNB1, DDIT3, DDX10, DDX6, DEK, DUSP22, EGFR, EIF4A2, ELF4, ELL, ELN, EML4, EP300, EPOR, EPS15, ERBB2, ERG, ETS1, ETV1, ETV4, ETV5, ETV6, EWSR1, FCGR2B, FCRL4, FEV, FGFR1, FGFR1OP, FGFR2, FGFR3, FLI1, FNBP1, FOXO1, FOXO3, FOXO4, FOXP1, FSTL3, FUS, GAS7, GLI1, GMPS, GPHN, HERPUD1, HEY1, HIP1, HIST1H4I, HLF, HMGA1, HMGA2, HOXA11, HOXA13, HOXA3,*
Fusions	
ABL1, AKT3, ALK, AXL, BRAF, EGFR, ERBB2, ERG, ETV1, ETV4, ETV5, FGFR1, FGFR2, FGFR3, MET, NTRK1, NTRK2, NTRK3, PDGFRA, PPARG, RAF1, RET, ROS1	

© 2019 Seligson ND et al. *JAMA Network Open.*

	HOXA9, HOXC11, HOXC13, HOXD11, HOXD13, HSP90AA1, HSP90AB1, IGH, IGK, IGL, IKZF1, IL21R, IL3, IRF4, ITK, JAK1, JAK2, JAK3, JAZF1, KAT6A, (MYST3), KDSR, KIF5B, KMT2A, (MLL), LASP1, LCP1, LMO1, LMO2, LPP, LYL1, MAF, MAFB, MALT1, MDS2, MECOM, MKL1, MLF1, MLLT1, (ENL), MLLT10, (AF10), MLLT3, MLLT4, (AF6), MLLT6, MN1, MNX1, MSI2, MSN, MUC1, MYB, MYC, MYH11, MYH9, NACA, NBEAP1, (BCL8), NCOA2, NDRG1, NF1, NF2, NFKB2, NIN, NOTCH1, NPM1, NR4A3, NSD1, NTRK1, NTRK2, NTRK3, NUMA1, NUP214, NUP98, NUTM2A, OMD, P2RY8, PAFAH1B2, PAX3, PAX5, PAX7, PBX1, PCM1, PCSK7, PDCD1LG2, (PDL2), PDE4DIP, PDGFB, PDGFRA, PDGFRB, PER1, PHF1, PICALM, PIM1, PLAG1, PML, POU2AF1, PPP1CB, PRDM1, PRDM16, PRRX1, PSIP1, PTCH1, PTK7, RABEP1, RAF1, RALGDS, RAP1GDS1, RARA, RBM15, RET, RHOH, RNF213, ROS1, RPL22, RPN1, RUNX1, RUNX1T1, (ETO), RUNX2, SEC31A, SEPT5, SEPT6, SEPT9, SET, SH3GL1, SLC1A2, SNX29, (RUNDC2A), SRSF3, SS18,

© 2019 Seligson ND et al. *JAMA Network Open.*

	SSX1, SSX2, SSX4, STAT6, STL, SYK, TAF15, TAL1, TAL2, TBL1XR1, TCF3, (E2A), TCL1A, (TCL1), TEC, TET1, TFE3, TFG, TFPT, TFRC, TLX1, TLX3, TMPRSS2, TNFRSF11A, TOP1, TP63, TPM3, TPM4, TRIM24, TRIP11, TTL, TYK2, USP6, WHSC1, (MMSET, or, NSD2), WHSC1L1, YPEL5, ZBTB16, ZMYM2, ZNF384, ZNF521

© 2019 Seligson ND et al. *JAMA Network Open.*

eTable 2: Description of Pathways

etable 2: Description of Pathways						
Cell Cycle Regulation	DNA Damage Repair	Epigenetics		Growth Signaling		Other
CCND3	*ABL1*	*APC*	*MED12*	*ADGRA2*	*INPP5D*	*BCOR*
CDKN2A	*BAP1*	*ATRX*	*PCBP1*	*ALK*	*LRRK2*	*CASP8*
CDKN2B	*BARD1*	*AXIN1*	*RARA*	*AR*	*MAP2K2*	*CIITA*
DKC1	*BRCA1*	*BCORL1*	*RPS19*	*ASXL1*	*MAP3K1*	*CRBN*
MEF2B	*BRCA2*	*EED*	*RUNX1*	*CCT6B*	*MAP3K13*	*ECT2L*
MKI67	*CHEK2*	*HIST1H1E*	*SETBP1*	*CD36*	*MYO18A*	*FAS*
NUP98	*FANCA*	*JARID2*	*SMARCA4*	*CPS1*	*NCOR1*	*FAT3*
POT1	*FANCL*	*KDM4C*	*TAF1*	*CSF1R*	*NCOR2*	*FBXO31*
RB1	*MLH1*	*KDM5C*	*TCF3*	*DDX3X*	*NOTCH1*	*FBXW7*
RELN	*MSH2*	*KMT2A*	*TERT*	*DTX1*	*NOTCH2*	*GATA3*
SGK1	*MSH3*	*KMT2C*	*TET2*	*EPHA3*	*NOTCH3*	*IKZF3*
SMC1A	*MUTYH*	*KMT2D*	*TOP1*	*EPHB1*	*NTRK1*	*KEAP1*
	PMS2	*LRP1B*	*YY1AP1*	*ERBB2*	*PASK*	*NFKBIA*
	RAD50	*TET2*	*ZNF217*	*FGF4*	*PC*	*NOD1*
	RAD51C		*ZNF703*	*GNAS*	*PIK3CA*	*PDCD1LG2*
				HGF	*PTCH1*	*TP63*
				HSP90	*RICTOR*	*TRAF3*
				ICK	*ROS1*	

© 2019 Seligson ND et al. *JAMA Network Open.*

				IDH1<br>*IGF1R*	*TSC1*<br>*TSC2*<br>*TYK2*	

© 2019 Seligson ND et al. *JAMA Network Open.*

eFigure 1: Distribution of Genomic *WWTR1-CAMTA1* Fusion Expression

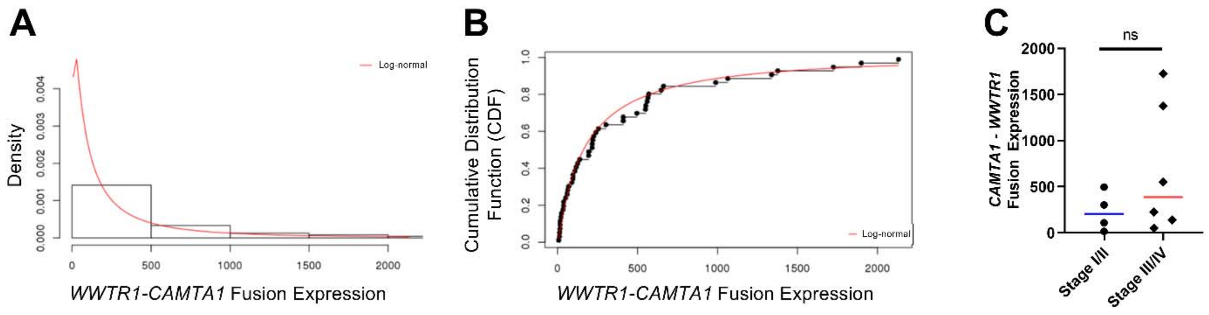

A,B) Fusion expression data from 45 subjects with *WWTR1-CAMTA1* fusion data available. *WWTR1-CAMTA1* fusion expression demonstrated a right-skewed, log-normal distribution (**A**) corroborated by the plot between empirical and theoretical cumulative distribution function (CDF; **B**). **C**) In a clinical cohort of 10 subjects with *WWTR1-CAMTA1* fusion data available, subjects with stage III/IV disease demonstrated a non-significant trend towards increased expression of the *WWTR1-CAMTA1* fusion (677±706 vs 231±213 copies; p=0.2).

© 2019 Seligson ND et al. *JAMA Network Open.*

eTable 3: Gene Variants in EHE

etable 3: Gene Variants in EHE		
Gene	Description	Variant Call
ABL1	A1102V	Unknown Pathogenicity
ADGRA2	P1115_S1116insTGEGPP	Unknown Pathogenicity
ALK - ALK	deletion	Unknown Pathogenicity
APC	Q1045*	Known Pathogenicity
APC	I1307K	Known Pathogenicity
APC	3920T>A	Known Pathogenicity
APC	Q1549K	Unknown Pathogenicity
AR	G457_G460del	Unknown Pathogenicity
ASXL1	S214N	Unknown Pathogenicity

© 2019 Seligson ND et al. *JAMA Network Open.*

ASXL1	M1492V	Unknown Pathogenicity
ATM	Amplification	Known Pathogenicity
ATRX	S1913*	Known Pathogenicity
ATRX	E225*	Known Pathogenicity
ATRX	H475D	Unknown Pathogenicity
AXIN1	R723*	Unknown Pathogenicity
BAP1	P147L	Unknown Pathogenicity
BARD1	F387L	Unknown Pathogenicity
BCOR	L722F	Unknown Pathogenicity
BCOR	T1696M	Unknown Pathogenicity
BCORL1	S1543L	Unknown Pathogenicity

© 2019 Seligson ND et al. *JAMA Network Open.*

BRCA1	H839fs*7	Known Pathogenicity
BRCA2	S2887T	Unknown Pathogenicity
CAMTA1 - WWTR1	fusion	Known Pathogenicity
CASP8	K384Q	Unknown Pathogenicity
CASP8	M1?	Unknown Pathogenicity
CCND3	R93*	Unknown Pathogenicity
CCT6B	splice site 615-2A>G	Known Pathogenicity
CD36	M156I	Unknown Pathogenicity
CD36	D244N	Unknown Pathogenicity
CDKN2A	R80*	Known Pathogenicity
CDKN2A	P81L	Known Pathogenicity

© 2019 Seligson ND et al. *JAMA Network Open.*

CDKN2A	deletion	Known Pathogenicity
CDKN2A	Amplification	Known Pathogenicity
CDKN2A	G101R	Unknown Pathogenicity
CDKN2B	deletion	Known Pathogenicity
CHEK2	T367fs*15	Known Pathogenicity
CIITA	V418A	Unknown Pathogenicity
CIITA	A1032V	Unknown Pathogenicity
CPS1	A347S	Known Pathogenicity
CRBN	I154R	Unknown Pathogenicity
CSF1R	V117A	Unknown Pathogenicity
DDX3X	A502T	Known Pathogenicity

© 2019 Seligson ND et al. *JAMA Network Open.*

DDX3X	I166F	Unknown Pathogenicity
DKC1	K505_A506insK	Unknown Pathogenicity
DTX1	Q291K	Unknown Pathogenicity
ECT2L	R852Q	Known Pathogenicity
EED	M15V	Unknown Pathogenicity
EPHA3	C259*	Unknown Pathogenicity
EPHB1	D602E	Unknown Pathogenicity
ERBB2	R896H	Known Pathogenicity
FANCA	M160I	Known Pathogenicity
FANCA	splice site 3828+1G>C	Known Pathogenicity
FANCL	C132R	Unknown Pathogenicity

© 2019 Seligson ND et al. *JAMA Network Open.*

FAS	E167K	Unknown Pathogenicity
FAT3	V2757I	Unknown Pathogenicity
FAT3	R258H	Unknown Pathogenicity
FAT3	Y221C	Unknown Pathogenicity
FBXO31	P524L	Unknown Pathogenicity
FBXW7	Amplification	Known Pathogenicity
FGF4	Y166C	Unknown Pathogenicity
GATA3	359 C>T	Unknown Pathogenicity
GNAS	Q332E	Unknown Pathogenicity
GNAS	T415_G423del	Unknown Pathogenicity
HGF	I306N	Unknown Pathogenicity

© 2019 Seligson ND et al. *JAMA Network Open.*

HIST1H1E	K177_A184del	Unknown Pathogenicity
HSP90AA1	E365_E366insEK	Unknown Pathogenicity
HSP90AA1	G7W	Unknown Pathogenicity
ICK	Q240H	Unknown Pathogenicity
IDH1	R119Q	Known Pathogenicity
IGF1R	D675N	Unknown Pathogenicity
IKZF3	R458C	Unknown Pathogenicity
INPP5D	F846S	Unknown Pathogenicity
JARID2	P139T	Unknown Pathogenicity
KDM4C	A305S	Unknown Pathogenicity
KDM5C	P1429L	Known Pathogenicity

© 2019 Seligson ND et al. *JAMA Network Open.*

KEAP1	F478fs*2	Known Pathogenicity
KMT2A	P1767fs*53	Unknown Pathogenicity
KMT2C	D2201H	Known Pathogenicity
KMT2C	A374V	Unknown Pathogenicity
KMT2C	P840H	Unknown Pathogenicity
KMT2D	R4238H	Unknown Pathogenicity
LRP1B	R4363G	Unknown Pathogenicity
LRRK2	V2495I	Known Pathogenicity
LRRK2	M2155L	Unknown Pathogenicity
LRRK2	C2154F	Unknown Pathogenicity
MAP2K2	I14S	Unknown Pathogenicity

© 2019 Seligson ND et al. *JAMA Network Open.*

MAP3K1	V569I	Known Pathogenicity
MAP3K13	D601A	Unknown Pathogenicity
MED12	Q2076_Y2077insQ	Unknown Pathogenicity
MEF2B	L251F	Unknown Pathogenicity
MKI67	E1487V	Unknown Pathogenicity
MKI67	I2958T	Unknown Pathogenicity
MKI67	D751E	Unknown Pathogenicity
MLH1	E605*	Known Pathogenicity
MLH1	R9G	Unknown Pathogenicity
MSH2	E358D	Unknown Pathogenicity
MSH3	K255*	Known Pathogenicity

© 2019 Seligson ND et al. *JAMA Network Open.*

MSH3	A57_A62del	Unknown Pathogenicity
MUTYH	G382D	Known Pathogenicity
MYO18A	A1314T	Unknown Pathogenicity
NCOR1	V2293M	Unknown Pathogenicity
NCOR2	E859A	Unknown Pathogenicity
NCOR2	A775T	Unknown Pathogenicity
NFKBIA	amplification	Known Pathogenicity
NOD1	F515fs*228	Known Pathogenicity
NOD1	A829G	Unknown Pathogenicity
NOTCH1	3835C>T	Known Pathogenicity
NOTCH1	R1132C	Unknown Pathogenicity

© 2019 Seligson ND et al. *JAMA Network Open.*

NOTCH2	G2291W	Unknown Pathogenicity
NOTCH3	R75W	Unknown Pathogenicity
NTRK1	splice	Unknown Pathogenicity
NUP98	E948D	Unknown Pathogenicity
PASK	G327S	Unknown Pathogenicity
PC	M1145I	Unknown Pathogenicity
PCBP1	T91I	Unknown Pathogenicity
PDCD1LG2	T217S	Unknown Pathogenicity
PIK3CA	N515S	Known Pathogenicity
PIK3CA	R949Q	Unknown Pathogenicity
PMS2	S46N	Known Pathogenicity

© 2019 Seligson ND et al. *JAMA Network Open.*

POT1	D617fs*9	Known Pathogenicity
PTCH1	G51del	Unknown Pathogenicity
RAD50	E1232A	Unknown Pathogenicity
RAD51C	R24Q	Unknown Pathogenicity
RARA	T179M	Unknown Pathogenicity
RB1	R320*	Known Pathogenicity
RB1	deletion	Known Pathogenicity
RB1	G203W	Unknown Pathogenicity
RELN	T1916A	Unknown Pathogenicity
RELN	L1732F	Unknown Pathogenicity
RELN	G937A	Unknown Pathogenicity

RELN	K3100del	Unknown Pathogenicity
RICTOR	P1397H	Unknown Pathogenicity
ROS1	G1196R	Known Pathogenicity
ROS1	R2269*	Unknown Pathogenicity
ROS1	E2244Q	Unknown Pathogenicity
RPS19	R102Q	Unknown Pathogenicity
RUNX1	A187T	Unknown Pathogenicity
SETBP1	R11Q	Unknown Pathogenicity
SGK1	R12Q	Unknown Pathogenicity
SMARCA4	splice site 4911+1G>A	Known Pathogenicity
SMARCA4	K892R	Unknown Pathogenicity

© 2019 Seligson ND et al. *JAMA Network Open.*

SMC1A	S1188C	Unknown Pathogenicity
TAF1	K424R	Unknown Pathogenicity
TCF3	M415V	Unknown Pathogenicity
TERT	Amplification	Known Pathogenicity
TET2	100C>T	Known Pathogenicity
TOP1	I133V	Unknown Pathogenicity
TP63	N467S	Unknown Pathogenicity
TRAF3	V47M	Unknown Pathogenicity
TSC1	3185G>A	Known Pathogenicity
TSC2	R1159Q	Unknown Pathogenicity
TSC2	K351R	Unknown Pathogenicity

© 2019 Seligson ND et al. *JAMA Network Open.*

TYK2	R281S	Unknown Pathogenicity
TYK2	M8L	Unknown Pathogenicity
YY1AP1	C42_R45>C	Unknown Pathogenicity
ZNF217	N55fs*31	Unknown Pathogenicity
ZNF703	amplification	Known Pathogenicity
ZNF703	H402_D403>PTHLGGSSCSTCSAHD	Unknown Pathogenicity

© 2019 Seligson ND et al. *JAMA Network Open.*

eFigure 2: Age At Diagnosis

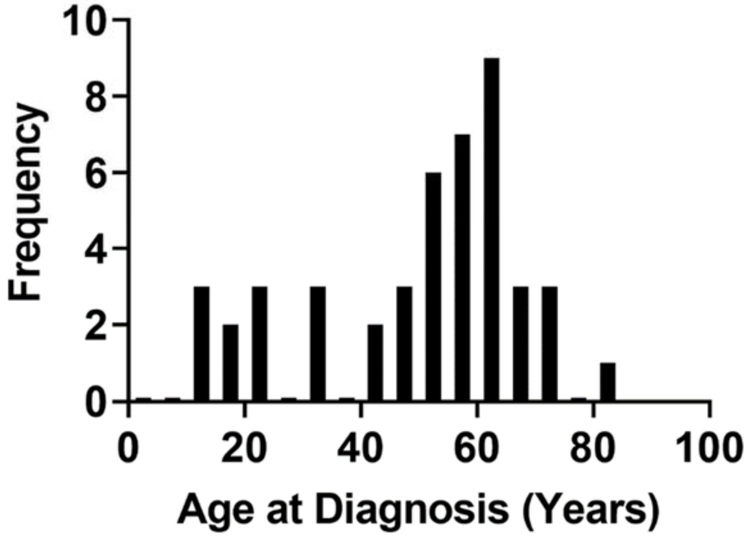

In 45 EHE subjects with data available, mean age at diagnosis was 50.2 years of age with an apparent bimodal distribution around the 45 year mark.

© 2019 Seligson ND et al. *JAMA Network Open.*

eMethods: Models

Utilizing the mclust package in Rv.3.4.3, a Gaussian finite mixture model was fit to the data. A second forced a one-compartment model was then fit to the same data. These two models were then tested by calculating the difference in model fit by a log-likelihood ratio test (Log-likelihood one-component model -207.2 vs two-component model -197.6, difference 9.6, 95%CI 0.0-23.8, p=0.02). The 95% confidence interval (95%CI) was generated by bootstrapping the model comparison for 1,000 iterations. Statistical significance here suggests the distribution of the tested data is significantly different than a one-compartment model distribution.

© 2019 Seligson ND et al. *JAMA Network Open.*

eTable 4: Clinicogenomic Features of *CDKN2A/B* Variant Subjects

etable 4: Clinicogenomic Features of *CDKN2A/B* Variant Subjects							
Case	#1	#2	#3	#4	#5	#6	#7
Age at Diagnosis (year)	51	56	11	50	53	70	65
Gender	Female	Male	Female	Female	Male	Male	Female
TMB	2.4	0.8	0.8	0.8	0	0.8	2.4
Microsatellite Instability (MSI)	Stable	Stable	Stable	N/A	N/A	Stable	Stable
WWTR1-CAMTA1 Expression	566	63	69	97	194	550	1376
CDKN2A	R80*	P81L	G101R	Deletion	Deletion	Deletion	Deletion
CDKN2B	-	-	-	Deletion	Deletion	Deletion	Deletion
APC							Q1549K
ATRX			H475D				

© 2019 Seligson ND et al. *JAMA Network Open.*

AXIN1						R723*	
BARD1	F387L						
BCOR	L722F						
BRCA1	H839fs*7						
BRCA2	S2887T						
DDX3X							I166F
FANCA							M160I
FAS	E167K						
FAT3							Y221C
HIST1H1E							K177_A184del
IDH1							R119Q
MKI67				E1487V			
MSH3	K255*; A57_A62del						
NCOR2				E859A			
NFKBIA							Amplification
SMARCA4		K892R					
TP63						N467S	
TSC2				R1159Q			

© 2019 Seligson ND et al. *JAMA Network Open.*

YY1AP1				C42_R45<br>>C			

© 2019 Seligson ND et al. *JAMA Network Open.*

eTable 5: Targetable Genomic Variants

etable 5: Targetable Genomic Variants			
Gene	Subjects With Alteration (%)		OncoKB Level of Evidence
	Know Variant	VUS	
ROS1	1 (2%)	2 (4.1%)	Level 1
TSC1	1 (2%)	0 (0%)	Level 1
TSC2	0 (0%)	2 (4.1%)	Level 1
NTRK1	0 (0%)	1 (2%)	Level 1
BRCA1	1 (2%)	0 (0%)	Level 1
BRCA2	0 (0%)	1 (2%)	Level 1
ERBB2	1 (2%)	0 (0%)	Level 1
IDH1	1 (2%)	0 (0%)	Level 1
PTCH1	0 (0%)	1 (2%)	Level 3
CDKN2A	6 (12.2%)	1 (2%)	Level 4
Total	9 (18.4%)	6 (12.2%)	

© 2019 Seligson ND et al. *JAMA Network Open.*
